# Regular Aerobic, Resistance, and Cross-Training Exercise Prevents Reduced Vascular Function Following a High Sugar or High Fat Mixed Meal in Young Healthy Adults

**DOI:** 10.3389/fphys.2018.00183

**Published:** 2018-03-08

**Authors:** Emon K. Das, Pui Y. Lai, Austin T. Robinson, Joan Pleuss, Mohamed M. Ali, Jacob M. Haus, David D. Gutterman, Shane A. Phillips

**Affiliations:** ^1^Department of Medicine, Cardiovascular Center and Clinical Research Center, Medical College of Wisconsin, Milwaukee, WI, United States; ^2^Department of Physical Therapy, University of Illinois, Chicago, IL, United States; ^3^Department of Kinesiology and Nutrition, University of Illinois, Chicago, IL, United States; ^4^Integrative Physiology Laboratory, University of Illinois, Chicago, IL, United States; ^5^Department of Medicine, University of Illinois, Chicago, IL, United States

**Keywords:** regular exercise, vasodilation, dietary fats, dietary carbohydrates, endothelium, vascular, arteriosclerosis, postprandial lipemia

## Abstract

The postprandial state can negatively influence flow mediated dilation (FMD), a predictor of atherosclerosis and cardiovascular disease. This investigation was designed to determine the effect of regular aerobic and/or resistance exercise on postprandial FMD after a high sugar or high fat mixed meal. Forty-five healthy participants were recruited from one of four groups: lean sedentary (SED), runners, weight lifters, and cross-trainers. Participants were randomly crossed over to a high sugar meal (HSM) and a high fat mixed meal (HFMM; both fat and carbohydrate). Pre-and postprandial endothelial function was assessed for both meals using brachial artery FMD. Plasma lipids, insulin, glucose, hs-CRP, and SOD were also measured with both meals. Endothelium-independent dilation was determined via sublingual nitroglycerin. Brachial artery FMD was reduced in SED following the HSM (9.9 ± 0.9% at baseline, peak reduction at 60 min 6.5 ± 1.0%) and the HFMM (9.4 ± 0.9% at baseline, peak reduction at 120 min 5.9 ± 1.2%; *P* < 0.05 for both, Mean ± SEM). There was no change in FMD after either HSM or HFMM in runners, weight lifters, and cross-trainers. Post-prandial increases in blood glucose, insulin and triglycerides were less pronounced in the exercisers compared to SED. In addition, exercisers presented lower baseline plasma hs-CRP and higher SOD activity. Nitroglycerin responses were similar among groups. These results suggest that endothelial function is reduced in sedentary adults after a HSM or HFMM, but not in regular aerobic or resistance exercisers. This response may be due to favorable postprandial metabolic responses or lower postprandial levels of inflammation and oxidative stress. These findings may help to explain the cardioprotective effect of exercise.

## Introduction

Reduced endothelium dependent flow mediated dilation (FMD) precedes the development of atherosclerosis (Bonetti et al., 2003) and cardiovascular disease (CVD) (Higashi et al., 2009), and is a strong prognostic marker of long-term cardiovascular morbidity and mortality (Fichtlscherer et al., 2004). FMD is predominately mediated by the release of nitric oxide (NO), a gaseous compound with antiadhesive, antithrombotic, and vasodilatory properties (Joyner and Green, 2009). The effect of acute feeding has been well summarized (Thom et al., 2016). Both fat- and carbohydrate-rich foods generally regarded as healthful have not been shown to reduce FMD (e.g., walnuts or oatmeal) (Brock et al., 2006; Cortes et al., 2006; Karatzi et al., 2008). However, ingestion of meals high in refined sugars (Kawano et al., 1999; Title et al., 2000; Beckman et al., 2001; Ceriello et al., 2002, 2005; Weiss et al., 2008; Xiang et al., 2008; Mah et al., 2011) and added dietary fat (Plotnick et al., 1997; Vogel et al., 1997; Bae et al., 2001; Gaenzer et al., 2001; Ceriello et al., 2002, 2005; Padilla et al., 2006; Tushuizen et al., 2006; Tyldum et al., 2009; Credeur et al., 2015) have been shown to reduce FMD potentially through the induction of oxidative stress (Plotnick et al., 1997; Title et al., 2000; Bae et al., 2001; Tushuizen et al., 2006; Xiang et al., 2008). Thus, the postprandial state is thought to potentially facilitate atherogenesis (Zilversmit, 1979; Ceriello et al., 2002) through increased oxidative stress and reduction in NO bioavailability.

Regular exercise is associated with reductions in CVD morbidity and mortality (Arena et al., 2015) and improvements in endothelial function (Green et al., 2011). These findings are partially attributed to bolstered anti-oxidant defense systems (Ashor et al., 2015) which may improve NO bioavailability. In addition, acute exercise appears to be protective against postprandial reductions in vascular function. Performing acute endurance exercise several hours prior to consuming a high sugar meal prevents reduced endothelial function (Weiss et al., 2008). Postprandial FMD impairment following a high fat mixed meal is prevented by acute moderate intensity (Padilla et al., 2006) and high-intensity aerobic interval exercise (Tyldum et al., 2009). Acute resistance exercise prevents increased arterial stiffness following a high fat mixed meal (Augustine et al., 2014). Collectively, these findings indicate that regular exercise is beneficial for long-term cardiovascular health and that decrements in vascular function following a meal can be attenuated by acute exercise. However, the effects of regular exercise on postprandial endothelial function remain unexplored.

Therefore, the purpose of this study was to test the hypothesis that postprandial FMD is reduced following a high sugar or high fat mixed meal in sedentary adults, but maintained in individuals engaging in regular exercise. We also sought to determine if the type of exercise (resistance, aerobic, or cross-training) influenced postprandial FMD responses. We hypothesized that all exercisers would present favorable postprandial metabolic responses (e.g., glucose tolerance and insulin sensitivity), which may play a role in vascular responses (Augustine et al., 2014). Chronic exercise-induced prevention of diet-induced endothelial dysfunction might extend to protection against other lifestyle related activities shown to evoke endothelial dysfunction (Ghiadoni et al., 2000; Heiss et al., 2008; Phillips et al., 2011; Goslawski et al., 2013; Robinson et al., 2016), and explain some of the unaccounted for cardioprotective effects of exercise (Joyner and Green, 2009).

## Methods

### Study design and participants

All protocols were approved by the Institutional Review Boards at the Medical College of Wisconsin and the University of Illinois at Chicago. Fifty-two participants were enrolled and 45 completed the study. All subjects gave verbal and written informed consent in accordance with the Declaration of Helsinki. Participants underwent an initial screen for inclusion and exclusion criteria. Adults between the ages of 18 and 40 were recruited from one of four groups: (1) sedentary adults who had not regularly engaged in any exercise training programs for the previous 6 months; (2) runners who ran an average of 15–20 miles per week over the previous 6 months; (3) weight lifters who resistance trained with free weights or machines ≥3 times per week over the previous 6 months; and 4) cross-trainers who performed both resistance exercise ≥3 times per week and ran 15–20 miles per week for the previous 6 months. Participants were excluded if they had a history of CVD, hypertension [≥140/90 mmHg], hypercholesterolemia, eating disorders, diagnoses with diabetes or other endocrine disorders, pregnancy, tobacco or excessive alcohol use, or use of illicit drugs.

**Figure 1:**
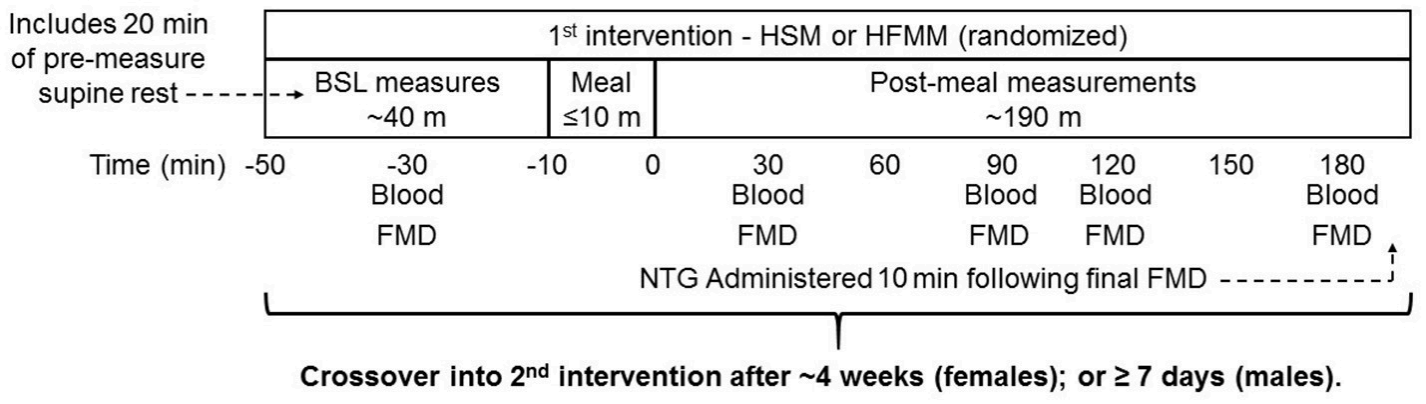
Study design: Sedentary participants (*n* = 11), runners (*n* = 12), weight lifters (*n* = 13), and cross-trainers (*n* = 9) were screened, consented, and enrolled in the study. Subjects underwent a resting FMD and blood draw followed by a 75 g glucose drink (high sugar meal; HSM) or a high fat mixed meal (HFMM) followed by repeated measures FMD and blood draws at 30, 60, 120, and 180 min post-meal. Participants were instructed to drink the meals within 10 min. Blood draws were taken for determination blood glucose, insulin, and triglycerides. Following ≥ 1-week washout for males and 1-month for females, the visit was repeated for the remaining meal. Meal order was randomized.

The study was a randomized, cross over design (Figure 1). Participants received the high sugar meal (HSM) and the high fat mixed meal (HFMM) in randomized order to prevent an order effect. There was a 7- to 30-day washout period between visits and a subset of participants (eight sedentary group, two runners, two weight lifters and one cross-trainer) returned for a third-time control visit where no meal was consumed. Women were tested in the early follicular phase of their menstrual cycle (i.e., days 1 through six via self-report).

### Meal composition

The HSM consisted of a solely carbohydrate 75 g glucose tolerance drink (orange flavored; NERL, Trutol, Fisher Scientific; total 300 kcal). There is no protein or fat in this beverage. The HFMM was given in the form of a shake that consisted of 8 fl. oz. Vanilla Resource 2.0 (Novartis), 150 g of ice cream (Vanilla Häagen Dazs®), and 7 g of dried egg yolk powder. The HFMM nutrient profile was as follows: 50 g of fat, 19 g of saturated fat, 339 mg of cholesterol, and 82 g of carbohydrate, 46 g of which were simple sugars. (total 910 kcal). Participants were instructed to consume each meal within 10 min. Several studies have used oral glucose tolerance test beverages (i.e., HSM) to elicit hyperglycemia induced decrements in FMD (Title et al., 2000; Ceriello et al., 2002; Xiang et al., 2008) and we designed the macronutrient composition of our HFMM to be similar to previous studies employing standardized HFMMs (Plotnick et al., 1997; Vogel et al., 1997).

### Measurement of flow- and nitroglycerin-mediated dilation

Brachial artery ultrasound assessment of FMD was performed with the participants in the supine position, using an 11-MHz probe (GE Logiq 500 pro-series) as previously described (Phillips et al., 2011). After the initial baseline ultrasound evaluation, participants transitioned to the reclining position and were given HSM or HFMM to consume within 10 min. Brachial artery FMD was then re-assessed in the supine position at 30, 60, 120, and 180 min post-ingestion. Participants were permitted to use the restroom between measures, however participants were supine for ≥10 min before each FMD evaluation. Nitroglycerin (NTG; 0.4 mg) was administered sublingually 10 min after the 180-min FMD at both visits. Ultrasound measurements were performed immediately before, as well as continuously throughout the 5 min after NTG administration. Shear rate (s^−1^) was calculated as blood velocity (cm/s) divided by vessel diameter as previously described (Phillips et al., 2011). The mean blood velocity was measured simultaneously in duplex mode with the brachial artery diameter. A single investigator performed the scans and a separate single investigator performed analysis while blinded to participant groups and meal condition (i.e., HSM or HFMM). Blood pressures were taken at the upper arm using an automated BP monitor (GE Dinamap) in the supine position with the lower arm supported by a pillow at the same time points at which FMD was performed.

### Blood profile measurements and analysis

Baseline blood samples were taken in the fasted condition (≥4 h) at baseline, and 30, 60, 120, and 180 min after the respective test meal. Plasma glucose concentration was measured using the glucose oxidase procedure (Beckman II autoanalyzer). Triglycerides (Stanbio), total cholesterol, and HDL- and direct LDL-cholesterol (Roche Diagnostics) were measured with spectrophotometric assays. The homeostasis model assessment for insulin resistance (HOMA-IR) was calculated as previously described (Matthews et al., 1985). The Matsuda insulin sensitivity index (ISI) was calculated as previously described to provide an estimate of whole body insulin sensitivity that also correlates with indices obtained from the hyperinsulinemic-euglycemic clamp (Matsuda and DeFronzo, 1999). Glucose and triglyceride area under the curve (AUC) for the 180 min following HSM and HFMM was calculated using the trapezoidal method to determine AUC (Le Floch et al., 1990) (GraphPad Prism Version 7.0; La Jolla, CA). Briefly, each time point measured is treated as a fraction of the total area. All time points are considered and combined for the total area which is computed using the trapezoid rule. We set our parameters such that baseline values were considered our theoretical “zero” and postprandial values lower than the physiological baseline are included in the model (i.e. they have a net “negative” effect on total area). High sensitivity C-reactive protein was measured with an enzyme immunoassay kit (MP Biomedicals). Superoxide dismutase (SOD) activity was assessed by measuring the dismutation of superoxide radicals generated by xanthine oxidase and hypoxanthine using a commercially available kit (Cayman Chemical).

### Statistics

Brachial artery diameter, percent brachial artery FMD, shear rate-normalized FMD, blood glucose, insulin, and triglycerides were assessed using two-way repeated-measures ANOVA (group × time). Clinical characteristics, CRP, and SOD, were assessed using one-way ANOVA. Where ANOVA showed a statistically significant difference (main effect or interaction), the Bonferroni-Sidak test for multiple comparisons was performed. For covariate testing, a generalized estimating equation (GEE) model was used to compare FMD change over time between groups. The same model with additional covariates (CRP, lipids, baseline BP, insulin, glucose, cholesterol, LDL, triglycerides, brachial artery diameter, shear rate, % body fat, BMI) in the model was used for testing if the covariates independently impacted the group effects on the FMD change over time. Mean ± SEM were reported and *P* < 0.05 was considered significant.

## Results

### Participant characteristics

Table 1 documents the characteristics of each of the groups. A total of 52 participants were screened and enrolled into the study. 45 participants completed all study requirements and were included in the final analysis. The mean age was 27 ± 1 years (Table 1). Participants in each group demonstrated normal profiles for blood pressure and blood lipids. While there was no difference in BMI or waist circumference among the groups, the sedentary participants had a significantly higher body fat percentage compared to the regular exercise groups (Table 1; *P* < 0.01). Plasma CRP was higher and SOD activity was lower in the SED adults compared to regular exercisers (Table 1). While CRP levels in the sedentary group appear to be relatively high, the reported values are well within the range of detection for the particular assay used. All the other parameters were not statistically different between groups.

**Table 1:** Data are presented as mean ± SEM.

**Group**	**Sedentary**	**Runners**	**Weight lifter**	**Cross-trainer**	***P*-Value**
Number, n (F/M)	11 (7/4)	12 (7/5)	13 (4/9)	9 (5/4)	–
Age (years)	27 ± 2	27 ± **1**	26 ± 1	26 ± 1	0.633
BMI (kg/m2)	27 ± 1	22 ± 0.4	24 ± 1	24 ± 1	0.076
% Body Fat	31 ± 2	21 ± 2^*^	23 ± 2^*^	22 ± 2^*^	**0.005**^†^
Waist circumference (cm)	81 ± 2	73 ± 1	81 ± 3	77 ± 3	0.061
Total cholesterol (mg/dl)	156 ± 4	147 ± 7	159 ± 15	161 ± 11	0.795
LDL (mg/dl)	103 ± 9	102 ± 7	86 ± 10	96 ± 6	0.410
HDL (mg/dl)	51 ± 4	46 ± 3	48 ± 5	54 ± 3	0.559
Glucose (mg/dL)	94 ± 2	93 ± 3	90 ± 2	93 ± 2	0.622
Insulin (μU/mL)	8.7 ± 2	8.0 ± 0.3	7.1 ± 1	6.4 ± 1	0.650
HOMA-IR	3.1 ± 0.1	2.7 ± 0.3	2.7 ± 0.1	2.7 ± 0.1	0.325
C-reactive protein (mg/L)	5.2 ± 1	1.8 ± 1^*^	0.9 ± 2^*^	1.2 ± 0.5^*^	<**0.001**^†^
SOD Activity (U/ml)	0.84 ± 0.1	1.2 ± 0.2^*^	1.2 ± 0.09^*^	1.4 ± 0.2^*^	**0.009**^†^

BMI, body mass index; HDL, high density lipoprotein; LDL, low density lipoprotein; SOD, superoxide dismutase.

† *Indicates significance for One-way ANOVA (P < 0.05)*.

* *Indicates significant difference observed vs. sedentary participants (P < 0.05)*.

### Vasodilator function

**Figure 2:**
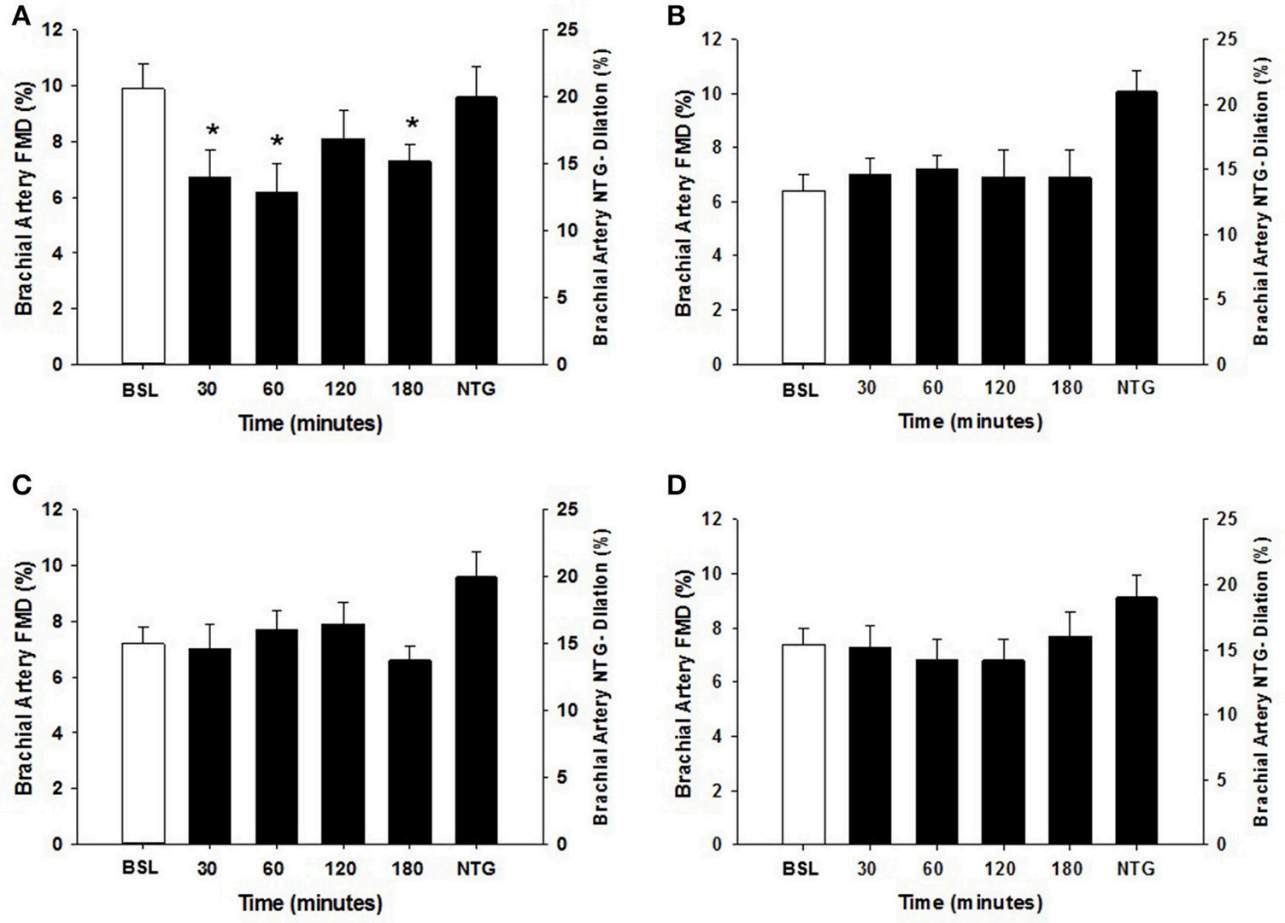
Brachial artery FMD responses to a 75 g glucose high sugar meal (HSM) at baseline, and 30, 60, 120, and 180 min post-ingestion in **(A)** sedentary participants (*n* = 11), **(B)** runners (*n* = 12), **(C)** weight lifters (*n* = 13), and **(D)** cross-trainers (*n* = 9). Brachial artery FMD scale is on the left Y-axis. Nitroglycerin (NTG) mediated dilation is depicted on the right Y-axis. All data presented as mean ± SEM. ^*^Indicates significant difference compared to baseline FMD measure (adjusted *P* < 0.05; Sidak-Bonferroni adjustment for ANOVA multiple comparisons).

**Figure 3:**
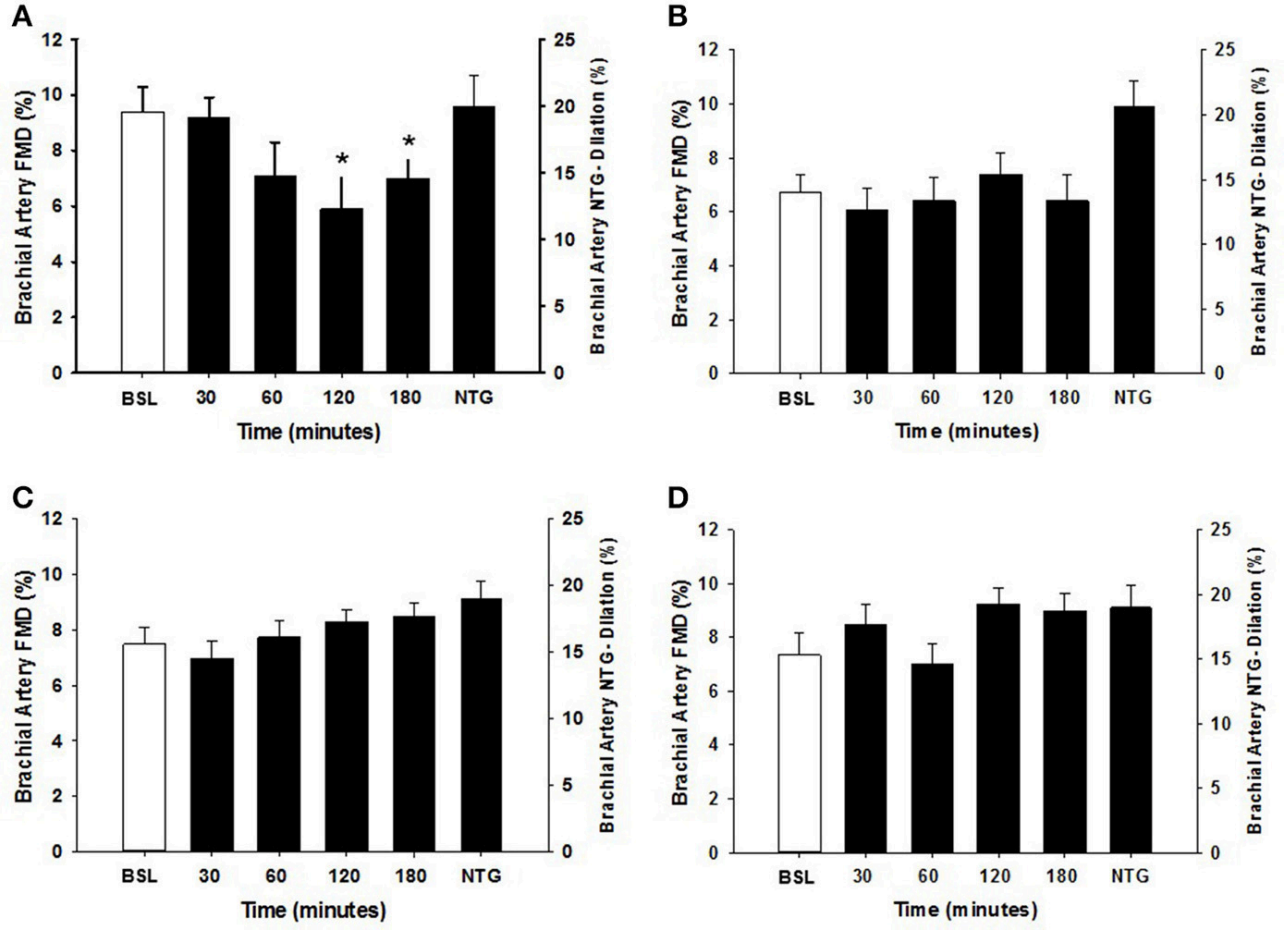
Brachial artery FMD responses to a high fat mixed meal (HFMM) at baseline, and 30, 60, 120, and 180 min post-ingestion in **(A)** sedentary participants (*n* = 11), **(B)** runners (*n* = 12), **(C)** weight lifters (*n* = 13), and **(D)** cross-trainers (*n* = 9). Brachial artery FMD scale is on the left Y-axis. Nitroglycerin (NTG) mediated dilation depicted on the right Y-axis. All data presented as mean ± SEM. ^*^Indicates significant difference compared to baseline FMD measure (adjusted *P* < 0.05; Sidak-Bonferroni adjustment for ANOVA multiple comparisons).

**Figure 4:**
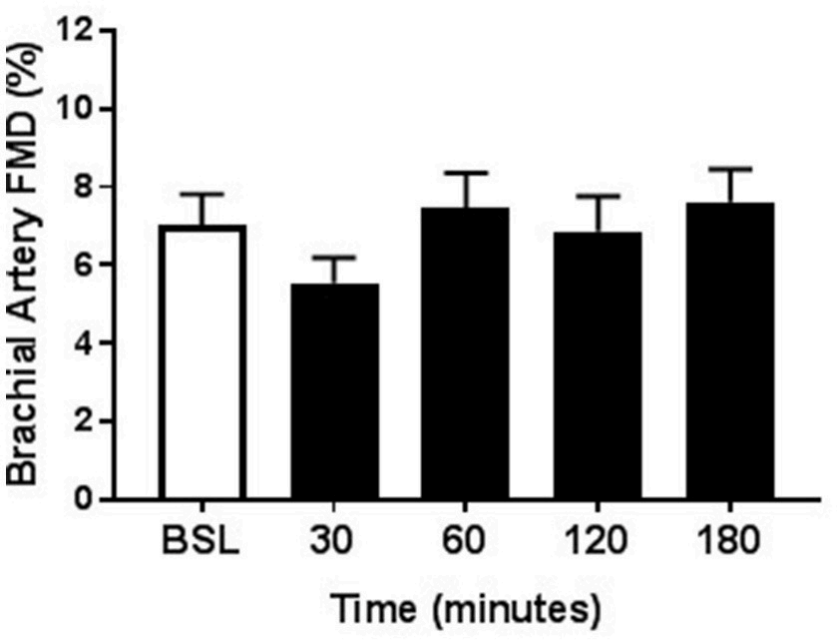
Repeated measures of Brachial Artery FMD. As time control, a subset of participants (*n* = 13) underwent the same protocol as the HSM and HFMM trials except they were given 12 oz. water. Brachial artery FMD responses at baseline, and 30, 60, 120, and 180 min post-ingestion are shown here. All data presented as mean ± SEM.

Within each group, the arterial diameter was similar across baseline and at each of the postprandial time points for both HSM and HFMM consumption (see Tables 2, 3). Consistent with previous studies in physically active populations (Padilla et al., 2006; Phillips et al., 2011; Robinson et al., 2016), the sedentary participants presented a greater FMD% at baseline (see Figures 2, 3). There was no group effect for brachial artery diameter during the HSM visit (see Table 2). There was a group effect for brachial artery diameter during the HFMM visit, but post hoc analysis did not reveal significant pairwise differences (Table 3). Regarding peak shear rate during hyperemia, which is thought to be a primary vasodilator stimulus (Pyke and Tschakovsky, 2007), there was no effect of time during the HSM or HFMM visits (Tables 2, 3). There was a group effect for peak shear rate during the HSM visit, but post hoc analysis did not reveal significant pairwise differences (Table 2). There was no group effect for peak shear rate during the HFMM visit (see Table 3). Brachial artery FMD was reduced in sedentary participants after HSM as indicated by a significant main effect for time, and for select pairwise comparisons (9.9 ± 0.9% pre-HSM to 6.8 ± 1.1% at 30 min, 6.5 ± 1.0% at 60 min, and 7.3 ± 0.6% at 180 min post-HSM; *P* < 0.05 compared to pre-HSM; Figure 2A). In contrast, there were no changes in FMD after HSM in any of the exercise trained groups (Figures 2B–D). Brachial artery FMD was also reduced in SED after HFMM, as indicated by a significant main effect for time, and for select pairwise comparisons (9.4 ± 0.9% pre-HFMM to 5.9 ± 1.2% at 120 min and 7.0 ± 0.8% at 180 min post-HFMM; *P* < 0.05 for both; Figure 3A). In contrast, there were no changes in FMD after HFMM in any of the exercise trained groups (Figures 3B–D). In a separate control study on a subset of our participants with no intervention, repeated FMD measures were similar at all-time points (Figure 4). There was no difference in endothelium-independent dilation to NTG between the groups after HSM or HFMM.

**Table 2:** Data presented as mean ± SEM.

	**Baseline**	**30 min**	**60 min**	**120 min**	**180 min**	***P*-value interaction time group**
**RESTING HR (bpm)**						
Sedentary	67 ± 2	72 ± 3	71 ± 3	73 ± 3	74 ± 3	0.9990
Runner	57 ± 3	56 ± 3^*^	58 ± 4^*^	60 ± 3^*^	57 ± 3^*^	0.3257
Weight lifter	57 ± 4	58 ± 4^*^	59 ± 3	60 ± 5^*^	60 ± 4^*^	<**0.0001^†^**
Cross-trainer	53 ± 3^*^	58 ± 4^*^	60 ± 4	61 ± 3	59 ± 3^*^	
**SYSTOLIC BP (mmHg)**						
Sedentary	124 ± 3	122 ± 4	119 ± 5	117 ± 5	121 ± 5	0.9991
Runner	120 ± 3	116 ± 2	113 ± 3	113 ± 3	110 ± 3	0.1837
Weight lifter	120 ± 3	116 ± 4	115 ± 4	113 ± 3	116 ± 4	**0.0003^†^**
Cross-trainer	112 ± 4	111 ± 5	108 ± 4	110 ± 4	109 ± 4	
**DIASTOLIC BP (mmHg)**						
Sedentary	54 ± 2	57 ± 2	57 ± 3	54 ± 3	56 ± 3	0.8154
Runner	57 ± 3	53 ± 2	54 ± 3	53 ± 2	53 ± 2	0.8198
Weight lifter	59 ± 2	55 ± 1	54 ± 1	56 ± 1	56 ± 1	0.4970
Cross-trainer	54 ± 1	54 ± 2	53 ± 2	56 ± 2	54 ± 2	
**BASELINE ARTERY DIAM (mm)**						
Sedentary	4.9 ± 0.3	4.9 ± 0.3	5.1 ± 0.3	5.1 ± 0.3	5.1 ± 0.3	0.9999
Runner	4.7 ± 0.3	4.7 ± 0.3	4.8 ± 0.3	4.9 ± 0.3	4.9 ± 0.3	0.9961
Weight lifter	5.3 ± 0.2	5.2 ± 0.2	5.3 ± 0.2	5.4 ± 0.2	5.4 ± 0.2	0.7456
Cross-trainer	4.9 ± 0.2	4.9 ± 0.2	4.9 ± 0.2	5.0 ± 0.2	5.0 ± 0.2	
**PEAK SHEAR RATE (s**^−1^**)**						
Sedentary	522 ± 50	575 ± 57	548 ± 46	513 ± 48	543 ± 63	0.9972
Runner	525 ± 63	524 ± 66	566 ± 60	574 ± 75	524 ± 49	0.7111
Weight lifter	465 ± 39	517 ± 36	543 ± 47	559 ± 56	511 ± 37	**0.0134^†^**
Cross-trainer	597 ± 47	585 ± 42	651 ± 36	633 ± 33	643 ± 51	

† Indicates significant difference observed vs. before glucose intake (P < 0.05).

* *Indicates significant difference vs. sedentary participants at each time. BP, blood pressure; FMD, flow-mediated dilation*.

**Table 3:** Data are presented as mean ± SEM.

	**Baseline**	**30 min**	**60 min**	**120 min**	**180 min**	***P*-value interaction time group**
**RESTING HR (bpm)**						
Sedentary	68 ± 2	77 ± 3	75 ± 3	75 ± 3	75 ± 3	0.9802
Runner	57 ± 2^*^	56 ± 2^*^	59 ± 2^*^	60 ± 3^*^	57 ± 2^*^	0.0669
Weight lifter	61 ± 3	63 ± 3^*^	66 ± 3	65 ± 4	66 ± 3	<**0.0001^†^**
Cross-trainer	53 ± 3^*^	58 ± 3^*^	60 ± 4^*^	61 ± 3^*^	59 ± 3^*^	
**SYSTOLIC BP (mmHg)**						
Sedentary	121 ± 3	121 ± 2	116 ± 2	113 ± 4	112 ± 5	0.9763
Runner	120 ± 3	116 ± 2	113 ± 3	113 ± 2	110 ± 2	**0.0034^†^**
Weight lifter	122 ± 6	120 ± 4	122 ± 4	108 ± 9	113 ± 3	0.7407
Cross-trainer	119 ± 3	116 ± 3	119 ± 3	112 ± 3	111 ± 2	
**DIASTOLIC BP (mmHg)**						
Sedentary	56 ± 1	56 ± 2	53 ± 2	53 ± 2	54 ± 2	0.9983
Runner	56 ± 3	54 ± 3	52 ± 3	51 ± 3	51 ± 3	0.1388
Weight lifter	60 ± 2	58 ± 1	58 ± 1	56 ± 1	56 ± 2	**0.0041^†^**
Cross-trainer	56 ± 1	55 ± 1	54 ± 1	55 ± 1	55 ± 1	
**BASELINE ARTERY DIAM (mm)**						
Sedentary	4.8 ± 0.2	4.8 ± 0.2	4.9 ± 0.2	5.1 ± 0.2	5.1 ± 0.2	0.9999
Runner	4.8 ± 0.4	4.8 ± 0.3	4.8 ± 0.3	5.0 ± 0.3	5.0 ± 0.3	0.4171
Weight lifter	5.3 ± 0.2	5.2 ± 0.2	5.3 ± 0.2	5.4 ± 0.2	5.5 ± 0.2	**0.0106^†^**
Cross-trainer	4.9 ± 0.2	4.8 ± 0.2	4.8 ± 0.2	5.0 ± 0.2	5.1 ± 0.2	
**PEAK SHEAR RATE (s**^−1^**)**						
Sedentary	522 ± 50	595 ± 82	548 ± 51	544 ± 51	545 ± 60	0.9784
Runner	548 ± 47	512 ± 53	580 ± 50	560 ± 50	585 ± 44	0.4542
Weight lifter	485 ± 37	519 ± 36	578 ± 44	555 ± 46	504 ± 39	0.1927
Cross-trainer	532 ± 42	575 ± 40	624 ± 39	635 ± 45	609 ± 43	

† Indicates significant difference observed vs. before high fat mixed meal (P < 0.05).

* *significant difference vs. sedentary participants at each time*.

### Hemodynamics and biochemical analyses

**Figure 5:**
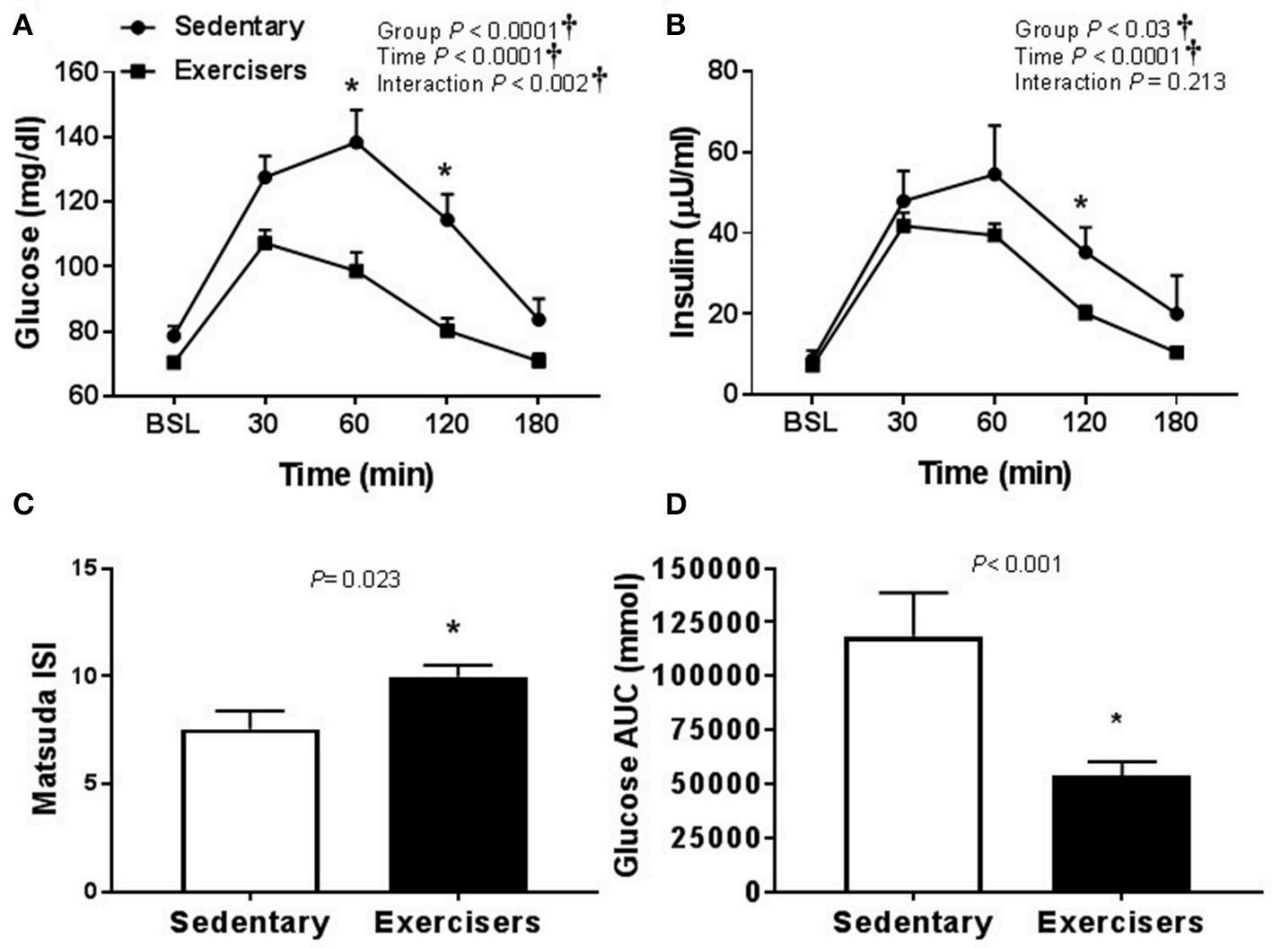
Glucose homeostatic responses to a 75 g glucose high sugar meal (HSM) at baseline, and 30, 60, 120, and 180 min post-ingestion in sedentary participants (*n* = 11) and exercisers (*n* = 33). **(A)** Blood glucose response over 180 min. **(B)** Serum insulin levels over 180 min. **(C)** Matsuda insulin sensitivity index (ISI) and **(D)** Glucose are under the curve (AUC) over the 180 min postprandial period. All data presented as mean ± SEM. ^†^Indicates significant main or interaction effect (*P* < 0.05). ^*^Indicates significant difference between sedentary participants and exercisers (adjusted *P* < 0.05; Sidak-Bonferroni adjustment for ANOVA multiple comparisons).

**Figure 6:**
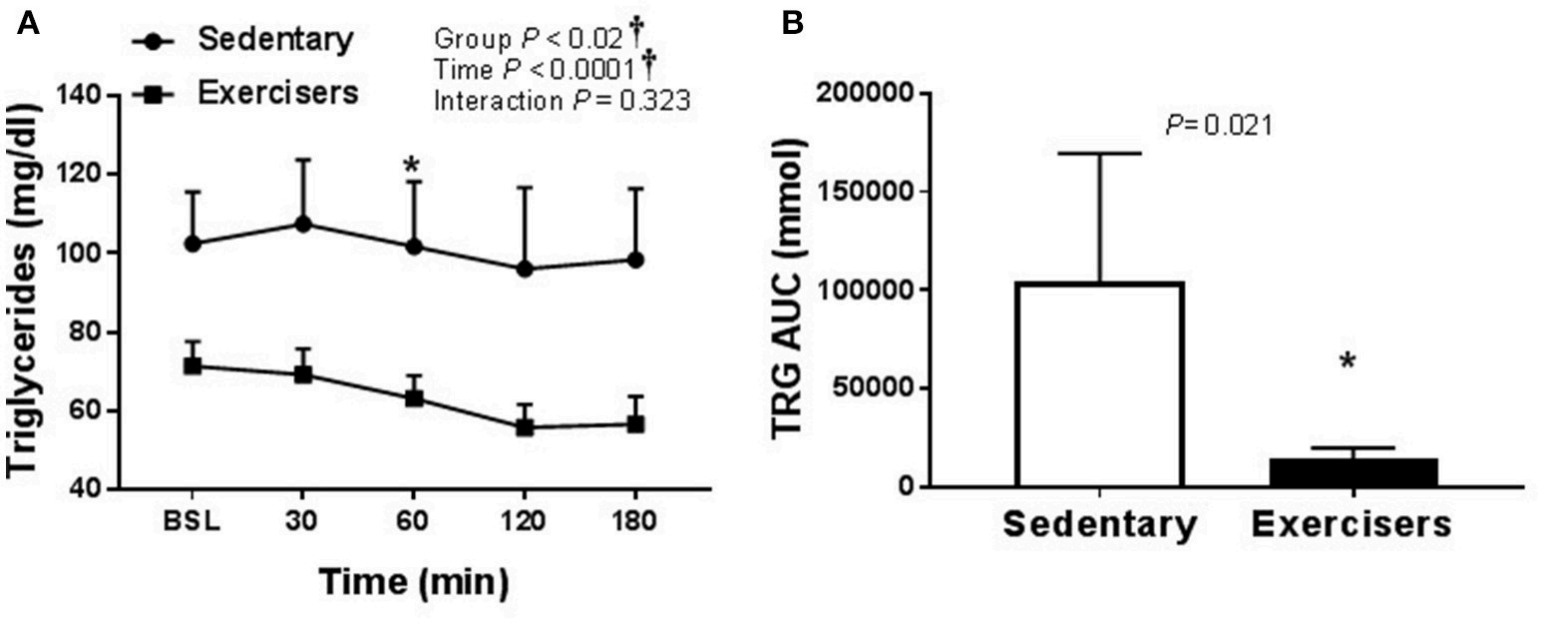
Serum triglycerides responses to a 75 g glucose high sugar meal (HSM) at baseline, and 30, 60, 120, and 180 min post-ingestion in sedentary participants (*n* = 11) and exercisers (*n* = 33). **(A)** Serum triglyceride levels over 180 min. and **(B)** Serum triglyceride area under the curve (AUC) over the 180 min postprandial period. All data presented as mean ± SEM. ^†^Indicates significant main or interaction effect (*P* < 0.05). ^*^Indicates significant difference between sedentary participants and exercisers (adjusted *P* < 0.05; Sidak-Bonferroni adjustment for ANOVA multiple comparisons).

There was no effect of HSM on SBP, but there was a significant group effect (Table 2). Post hoc analysis revealed no pairwise differences. There was a time effect of HFMM on SBP, but again no pairwise differences (Table 3). There was no effect of HSM on DBP and there were no group differences (Table 2). There was a significant group difference during the HFMM visit but no pairwise differences (Table 3). Fasting insulin and blood glucose levels were similar among all groups (Table 1). In a pooled analysis, glucose and insulin responses to HSM were increased in sedentary participants compared to exercisers (significant group and interaction effect; see Figures 5A,B). The Matsuda ISI was higher in exercisers, indicating greater insulin sensitivity (Figure 5C). Glucose AUC_0−180min_ was higher in SED participants compared to exercisers (Figure 5D). There was a significant group effect for postprandial triglycerides and triglyceride AUC_0−180min_ was higher in SED participants compared to exercisers following HSM (Figures 6A,B).

**Figure 7:**
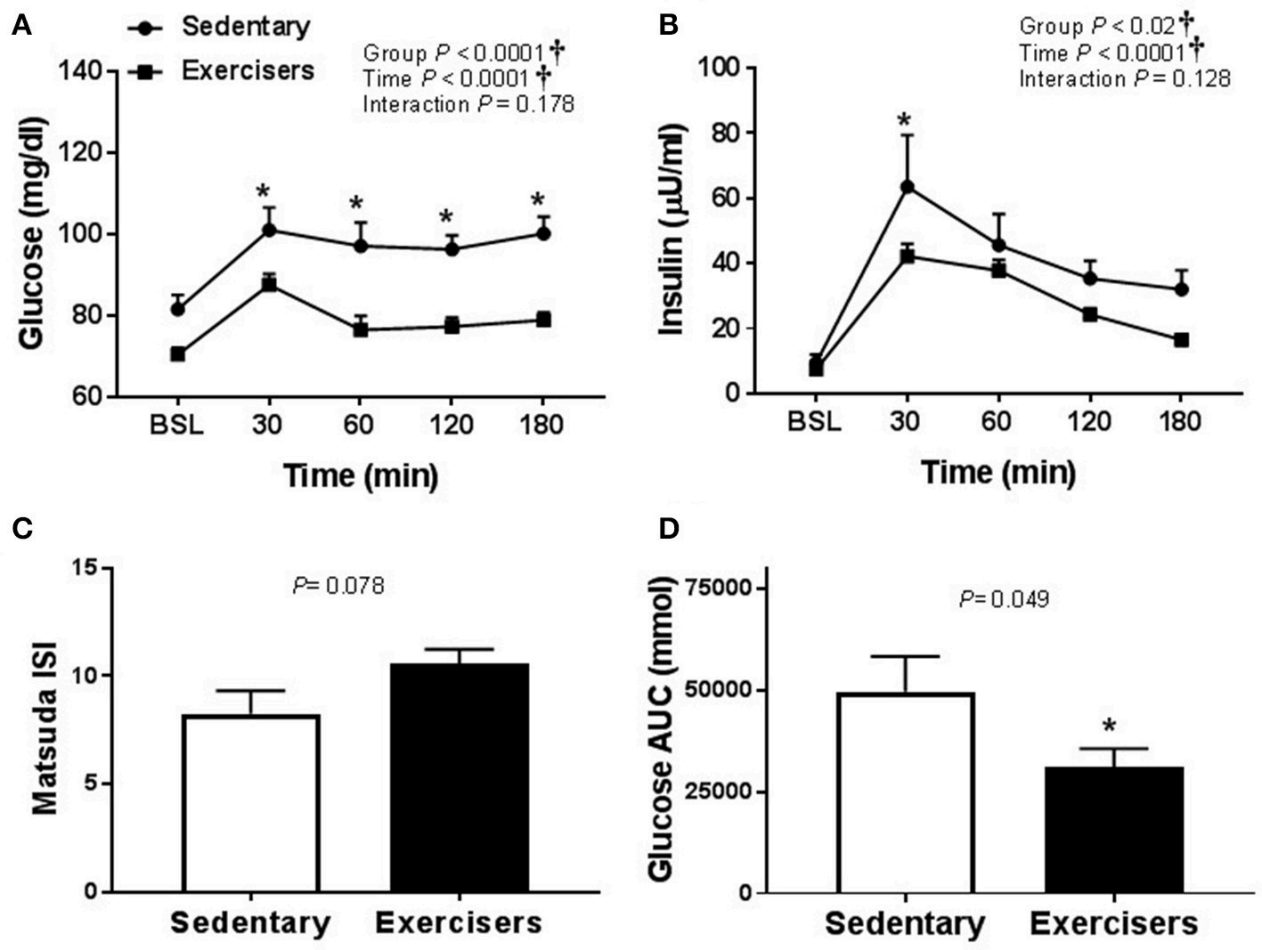
Glucose homeostatic responses to a high fat mixed meal (HFMM) at baseline, and 30, 60, 120, and 180 min post-ingestion in sedentary participants (*n* = 11) and exercisers (*n* = 33). **(A)** Blood glucose response over 180 min. **(B)** Serum insulin levels over 180 min. **(C)** Matsuda insulin sensitivity index (ISI) and **(D)** Glucose are under the curve (AUC) over the 180 min postprandial period. All data presented as mean ± SEM. ^†^Indicates significant main or interaction effect (*P* < 0.05). ^*^Indicates significant difference between sedentary participants and exercisers (adjusted *P* < 0.05 after Sidak-Bonferroni adjustment).

**Figure 8:**
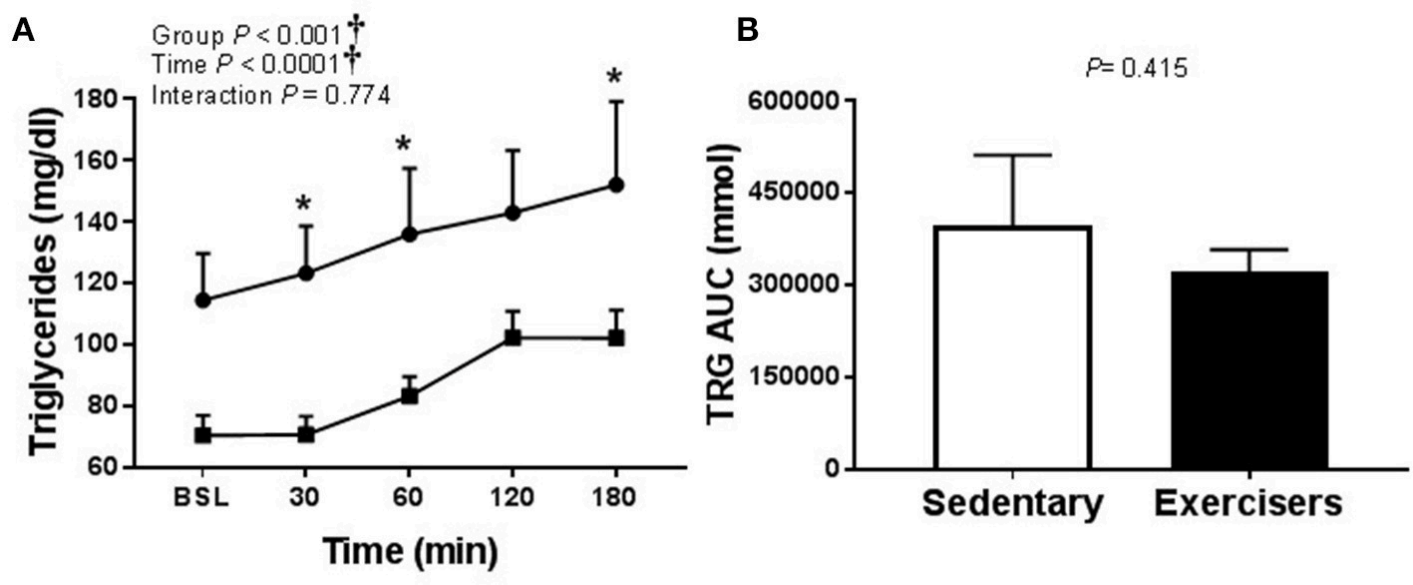
Serum triglyceride responses to a high fat mixed meal (HFMM) at baseline, and 30, 60, 120, and 180 min post-ingestion in sedentary participants (*n* = 11) and exercisers (*n* = 33). **(A)** Serum triglyceride levels over 180 min. and **(B)** Serum triglyceride area under the curve (AUC) over the 180 min postprandial period. All data presented as mean ± SEM. ^†^Indicates significant main or interaction effect (*P* < 0.05). ^*^Indicates significant difference between sedentary participants and exercisers (adjusted *P* < 0.05; Sidak-Bonferroni adjustment for ANOVA multiple comparisons).

Glucose and insulin concentrations were significantly greater in response to HFMM in SED participants compared to exercisers (significant group effect; see Figures 7A,B). In contrast to HSM, the Matsuda index was not different between sedentary participants compared to exercisers after HFMM, although there was a trend for elevated Matsuda index in the exercisers compared to SED participants (*P* = 0.078; Figure 7C). Glucose AUC_0−180min_ was higher in SED participants compared to exercisers following HFMM (*P* = 0.049; Figure 7D). There was a significant group effect for postprandial triglycerides (Figure 8A) but no difference between SED participants and exercisers triglyceride AUC_0−180min_ following HFMM (Figure 8B). The postprandial glycemia after HSM was greater than HFMM and postprandial lipemia after HFMM was greater than HSM.

**Figure 9:**
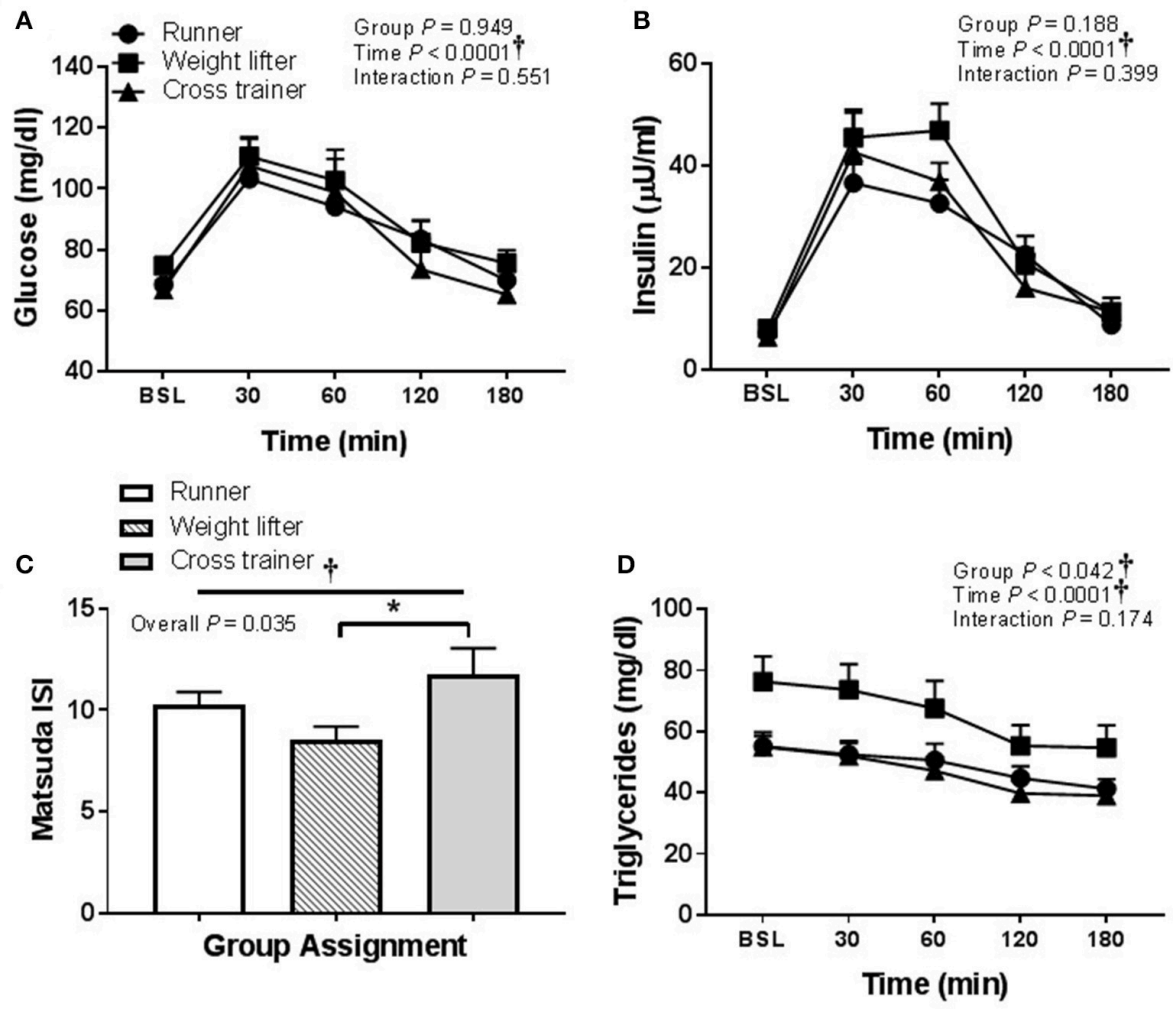
Metabolic responses at baseline, and 30, 60, 90, 120, and 180 min to a 75 g glucose high sugar meal (HSM) in runners (*n* = 11), weightlifters (*n* = 13), and cross-trainers (*n* = 9). **(A)** Blood glucose response over 180 min. **(B)** Serum insulin levels over 180 min **(C)** Matsuda insulin sensitivity index (ISI) and **(D)** Serum triglyceride levels over 180 min. All data presented as mean ± SEM. ^†^Indicates significant main or interaction effect (*P* < 0.05). ^*^Indicates significant difference between groups (adjusted *P* < 0.05; Sidak-Bonferroni adjustment for ANOVA multiple comparisons).

**Figure 10:**
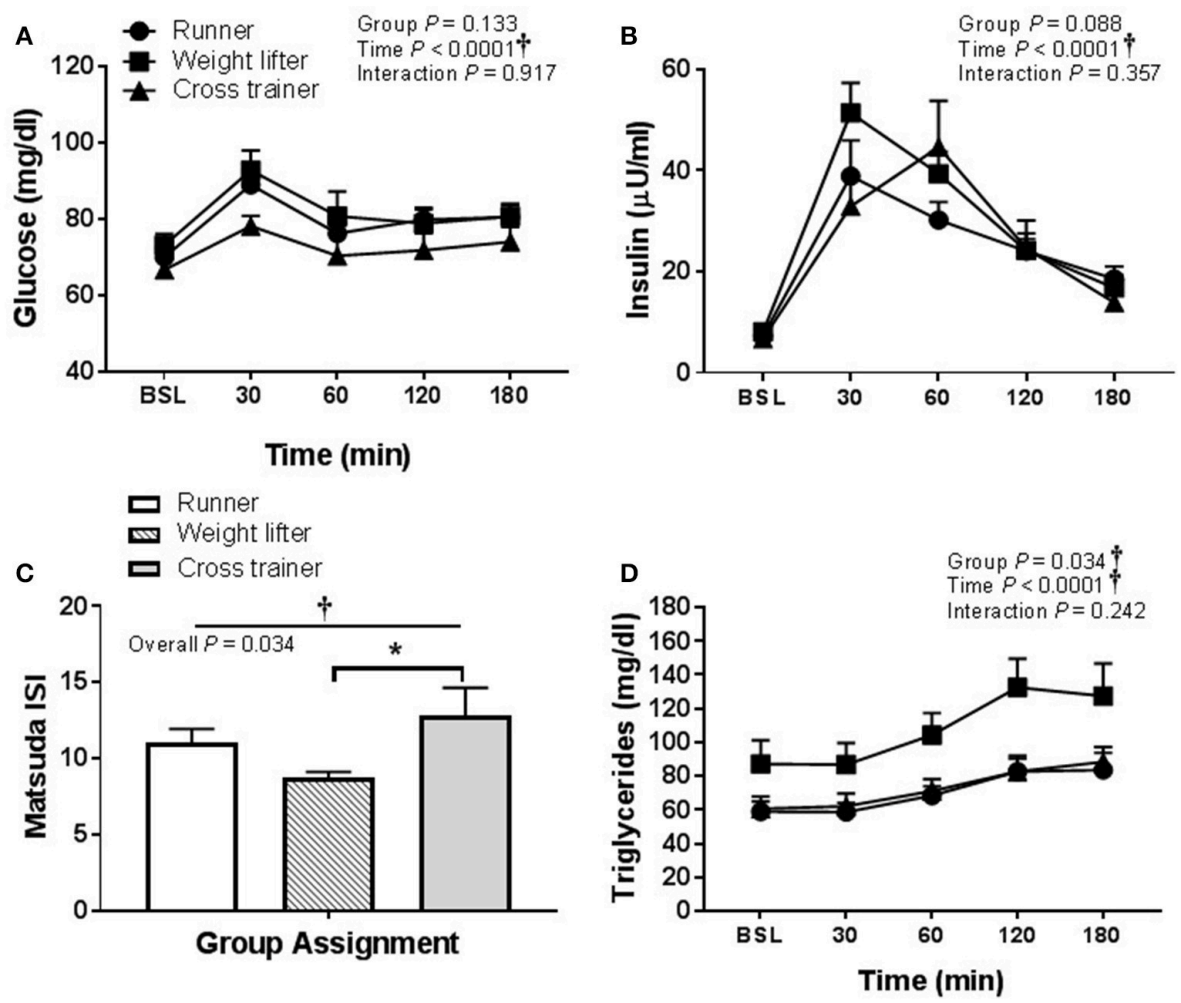
Metabolic responses at baseline, and 30, 60, 90, 120, and 180 min to a high fat mixed meal (HFMM) in runners (*n* = 11), weightlifters (*n* = 13), and cross-trainers (*n* = 9). **(A)** Blood glucose response over 180 min. **(B)** Serum insulin levels over 180 min. **(C)** Matsuda insulin sensitivity index (ISI) and **(D)** Serum triglyceride levels over 180 min. All data presented as mean ± SEM. ^†^Indicates significant main or interaction effect (*P* < 0.05). ^*^Indicates significant difference between groups (adjusted *P* < 0.05; Sidak-Bonferroni adjustment for ANOVA multiple comparisons).

In regard to pooling the exercisers' metabolic response data, glucose and insulin responses were similar between groups following HSM (Figures 9A,B) and HFMM (Figures 10A,B). However, weight lifters did exhibit lower insulin sensitivity following HSM compared to cross-trainers (Figure 9C) and there was a significant group effect for triglyceride responses to HSM (Figure 9D). Similarly, weight lifters exhibited lower insulin sensitivity following HFMM compared to cross-trainers (Figure 10C) and there was a significant group effect for triglyceride responses to HFMM (Figure 10D).

## Discussion

The primary findings of this study are that a single high sugar meal and high fat mixed meal reduce postprandial FMD in sedentary participants, but not in participants performing regular aerobic or resistance exercise. There was not a differential or a combined effect of aerobic and resistance exercise (i.e., cross-training) on postprandial FMD. Favorable metabolic responses to the feedings appear to have influenced the favorable postprandial FMD response in exercisers compared to sedentary participants. In addition, exercisers exhibited lower baseline plasma hs-CRP, a marker of inflammation, and higher SOD activity, an endogenous anti-oxidant compared to sedentary participants. Thus, exercisers may have been protected from postprandial glycemia- and lipemia-induced inflammation and oxidative stress ultimately resulting in a reduction in post-prandial FMD in SED participants.

We have previously demonstrated that endothelial dysfunction is associated with insulin resistance, suggesting that postprandial endothelial function may represent an important link in the development of insulin resistance (Mahmoud et al., 2016). Furthermore, postprandial hyperglycemia is a risk factor for CVD. Borderline high (100–140 mg/dl) postprandial glycemia has been shown to be one of the earliest signs of glucose dysregulation that precedes the development of atherosclerosis (Leiter et al., 2005). Consistent with this, we found that the postprandial glucose response to HSM and HFMM was increased in sedentary participants compared to regular exercisers. Since the magnitude of postprandial glycemia responses to high sugar were higher in SED participants, it is possible that the lower exposure to circulating glucose may explain the differences in postprandial FMD between SED participants and regular exercisers following HSM and HFMM. Title et al. (2000) demonstrated anti-oxidant treatment prevents decreased postprandial FMD without altering blood glucose following an oral 75 g glucose load in healthy participants. Collectively, these findings suggest that the greater postprandial glycemia in sedentary participants may have induced more vascular oxidative stress. Postprandial lipemia also may have been associated with excess oxidative stress.

Gaenzer et al. (2001) demonstrated impaired postprandial FMD following ingestion of a standardized fatty meal, and found that FMD correlated inversely with the magnitude of postprandial lipemia. In agreement, we found that sedentary participants had impaired postprandial FMD and had a larger postprandial elevation in triglycerides compared to the exercisers following both meals, particularly HFMM. SED participants' heightened levels of CRP and decreased SOD activity may have contributed to a potential interaction between increased postprandial lipemia, inflammation, and oxidative stress. *In vitro* studies have shown treatment of endothelial cells with free fatty acids impairs nitric oxide synthase production of NO through the inflammatory IKKβ/NFkβ pathway (Kim et al., 2005). Previous studies also indicate CRP plays a role in the activation of NFkβ (Jialal and Devaraj, 2009). High fat feedings in rodents have been shown to induce prolonged postprandial activation of inflammation and subsequent elevation in reactive oxygen species (Patel et al., 2007). Postprandial lipemia did not appear to impair the endothelial-independent dilations to NTG, as the reported values here are similar to our previous studies (Phillips et al., 2011; Robinson et al., 2016). This finding suggests that reduced NO bioavailability, rather than reduced NO sensitivity, contributes to the reduced FMD in the postprandial state. This finding is also in agreement with an elegant study conducted by Steinberg et al. (1997) whereby both exogenous and endogenous means of elevating free fatty acids in the human circulation resulted in suppression of endothelium-dependent, but not endothelium-independent induced dilation.

In conjunction with other studies showing that impaired endothelium function after a high sugar or high fat meal can be prevented by antioxidant treatment, our current findings support the notion that postprandial glycemia and lipemia reduce NO bioavailability in sedentary adults through an increase in oxidative stress (Plotnick et al., 1997; Title et al., 2000; Beckman et al., 2001; Xiang et al., 2008). Regular exercise may serve as a potent stimulus that results in adaptations that prevent such occurrences, such as improving insulin sensitivity and up-regulating endogenous anti-oxidant expression and/or activation (Rush et al., 2000; Robinson et al., 2017, 2018). Previous studies indicate that even short term inactivity can result in impaired FMD and insulin resistance (Hamburg et al., 2007; Boyle et al., 2013), while numerous studies indicate exercise improves insulin sensitivity (Borghouts and Keizer, 2000). Furthermore, exercise increases shear stress in the vasculature, which up-regulates antioxidant enzymes such as SOD and nitric oxide synthase expression (Rush et al., 2000; Hambrecht et al., 2003; Robinson et al., 2017, 2018). SOD promotes NO bioavailability by preventing superoxide induced scavenging of NO. Chronic exercise training also increases circulating levels of NO metabolites (Kingwell et al., 1997) suggesting that preservation of NO signaling in the face of oxidative stress may have been a mechanism by which FMD was preserved in exercisers, but not in the sedentary participants. We acknowledge resting FMD was lower in the exercise groups compared to the sedentary group and this is in agreement with previous studies (Tinken et al., 2008). It is thought that over time in the exercised vasculature functional changes in conduit arteries occur rapidly (i.e., improved FMD) and precede longer-term (at least several weeks) arterial remodeling *in vivo* (Green et al., 2012).

Limitations of this study include that training differences between sedentary participants and regular exercisers were not confirmed using aerobic exercise capacity (VO_2max_). Our participants were required to refrain from exercise for at least 24 h prior to study visits. However, repeated measures would need to be conducted to determine the duration from the last bout of acute exercise that regular exercise confers postprandial vascular protection. Without performing a glycemic and lipidemic clamps we were unable to determine if postprandial differences in FMD blood were solely attributable to the differences in postprandial glucose and lipids or if exercise induced upregulation in endogenous anti-oxidants were the primary mediators of preserved postprandial FMD in exercisers (i.e., exercisers experience less oxidative stress to a given amount of glucose or TRG exposure). We did not anticipate vascular smooth muscle dysfunction in our cohort given that the participants were young and healthy. Therefore, we did not have the participants take the NTG twice (i.e. pre- and postprandial). Postprandial NTG-mediated dilation was robust suggesting vascular smooth muscle function was not affected, however it was taken 3 h postprandial and that prevents us from concluding there was not vascular smooth muscle dysfunction in the more immediate postprandial period. Our sample size was not adequately powered to investigate potential sex or racial differences given the number of groups and time points. However, several other important considerations were made during interpretation, and highlight the strength of our findings. For instance, the resting baseline arterial diameter was not different between experimental groups, apart from the resistance exercise group appearing to have a slightly larger baseline diameter. The FMD baseline diameters and shear rates at postprandial time points after HSM and HFMM were also similar within groups, suggesting that changes in diameter or the shear stimulus for FMD were not responsible for any alterations observed in the postprandial FMD results (Pyke and Tschakovsky, 2007). To be expected, there were differences in body fat % observed between our sedentary participants and exercisers. We hypothesized this could potentially contribute to the differences in FMD responses to HSM or HFMM as more muscle mass may have contributed to greater insulin sensitivity.

### Perspectives

The results of this study indicate that flow-mediated dilation is maintained after a high sugar and a high fat mixed meal in individuals performing regular exercise. This protection does not appear to be dependent on the mode of exercise training (aerobic vs. resistance exercise vs. cross-training) indicating that these modalities afford similar protection of the endothelium against exposure to hyperglycemia and hyperlipidemia. It is known that exercise protects against CVD more so than would be predicted by a reduction in traditional risk factors alone (Joyner and Green, 2009). This preserved peripheral vascular function in the post-prandial state may be one mean through which regular exercise protects against insulin resistance and cardiovascular risk.

## Author contributions

ED, JP, DG, and SP designed the study. ED, PL, AR, and MA performed experiments. DG and SP provided materials. ED, PL, AR, JH, and SP performed analyses. ED, PL, AR, MA, JH, DG, and SP interpreted data. ED, AR, and SP wrote the manuscript. All authors edited and approved the manuscript.

### Conflict of interest statement

The authors declare that the research was conducted in the absence of any commercial or financial relationships that could be construed as a potential conflict of interest.

## Abbreviations

CRP: C-reactive protein
CVD: cardiovascular disease
FMD: flow mediated dilation
HFMM: high fat mixed meal
HSM: high sugar meal
NO: nitric oxide
NTG: nitroglycerin
SED: sedentary
SOD: Superoxide dismutase.

## References

[B1] Arena R. Guazzi M. Lianov L. Whitsel L. Berra K. Lavie C. J.. (2015). Healthy lifestyle interventions to combat noncommunicable disease-a novel nonhierarchical connectivity model for key stakeholders: a policy statement from the American Heart Association, European Society of Cardiology, European Association for Cardiovascular Prevention and Rehabilitation, and American College of Preventive Medicine. Eur. Heart J. 36, 2097–2109. 10.1093/eurheartj/ehv207 26138925

[B2] Ashor A. W. Lara J. Siervo M. Celis-Morales C. Oggioni C. Jakovljevic D. G.. (2015). Exercise modalities and endothelial function: a systematic review and dose-response meta-analysis of randomized controlled trials. Sports Med. 45, 279–296. 10.1007/s40279-014-0272-9 25281334

[B3] Augustine J. Tarzia B. Kasprowicz A. Heffernan K. S. (2014). Effect of a single bout of resistance exercise on arterial stiffness following a high-fat meal. Int. J. Sports Med. 35, 894–899. 10.1055/s-0033-1363266 24886920

[B4] Bae J. H. Bassenge E. Kim K. B. Kim Y. N. Kim K. S. Lee H. J.. (2001). Postprandial hypertriglyceridemia impairs endothelial function by enhanced oxidant stress. Atherosclerosis 155, 517–523. 10.1016/S0021-9150(00)00601-8 11254924

[B5] Beckman J. A. Goldfine A. B. Gordon M. B. Creager M. A. (2001). Ascorbate restores endothelium-dependent vasodilation impaired by acute hyperglycemia in humans. Circulation 103, 1618–1623. 10.1161/01.CIR.103.12.1618 11273987

[B6] Bonetti P. O. Lerman L. O. Lerman A. (2003). Endothelial dysfunction: a marker of atherosclerotic risk. Arterioscler. Thromb. Vasc. Biol. 23, 168–175. 10.1161/01.ATV.0000051384.43104.FC 12588755

[B7] Borghouts L. B. Keizer H. A. (2000). Exercise and insulin sensitivity: a review. Int. J. Sports Med. 21, 1–12. 10.1055/s-2000-8847 10683091

[B8] Boyle L. J. Credeur D. P. Jenkins N. T. Padilla J. Leidy H. J. Thyfault J. P.. (2013). Impact of reduced daily physical activity on conduit artery flow-mediated dilation and circulating endothelial microparticles. J. Appl. Physiol. 115, 1519–1525. 10.1152/japplphysiol.00837.2013 24072406 PMC3841822

[B9] Brock D. W. Davis C. K. Irving B. A. Rodriguez J. Barrett E. J. Weltman A.. (2006). A high-carbohydrate, high-fiber meal improves endothelial function in adults with the metabolic syndrome. Diabet. Care 29, 2313–2315. 10.2337/dc06-0917 17003313

[B10] Ceriello A. Assaloni R. Da Ros R. Maier A. Piconi L. Quagliaro L.. (2005). Effect of atorvastatin and irbesartan, alone and in combination, on postprandial endothelial dysfunction, oxidative stress, and inflammation in type 2 diabetic patients. Circulation 111, 2518–2524. 10.1161/01.CIR.0000165070.46111.9F 15867169

[B11] Ceriello A. Taboga C. Tonutti L. Quagliaro L. Piconi L. Bais B.. (2002). Evidence for an independent and cumulative effect of postprandial hypertriglyceridemia and hyperglycemia on endothelial dysfunction and oxidative stress generation: effects of short- and long-term simvastatin treatment. Circulation 106, 1211–1218. 10.1161/01.CIR.0000027569.76671.A8 12208795

[B12] Cortes B. Nunez I. Cofan M. Gilabert R. Perez-Heras A. Casals E. (2006). Acute effects of high-fat meals enriched with walnuts or olive oil on postprandial endothelial function. J. Am. Coll. Cardiol. 48, 1666–1671. 10.1016/j.jacc.2006.06.057 17045905

[B13] Credeur D. P. Holwerda S. W. Restaino R. M. King P. M. Crutcher K. L. Laughlin M. H.. (2015). Characterizing rapid-onset vasodilation to single muscle contractions in the human leg. J. Appl. Physiol. 118, 455–464. 10.1152/japplphysiol.00785.2014 25539935 PMC4329431

[B14] Fichtlscherer S. Breuer S. Zeiher A. M. (2004). Prognostic value of systemic endothelial dysfunction in patients with acute coronary syndromes: further evidence for the existence of the “vulnerable” patient. Circulation 110, 1926–1932. 10.1161/01.CIR.0000143378.58099.8C 15451794

[B15] Gaenzer H. Sturm W. Neumayr G. Kirchmair R. Ebenbichler C. Ritsch A.. (2001). Pronounced postprandial lipemia impairs endothelium-dependent dilation of the brachial artery in men. Cardiovasc. Res. 52, 509–516. 10.1016/S0008-6363(01)00427-8 11738068

[B16] Ghiadoni L. Donald A. E. Cropley M. Mullen M. J. Oakley G. Taylor M.. (2000). Mental stress induces transient endothelial dysfunction in humans. Circulation 102, 2473–2478. 10.1161/01.CIR.102.20.2473 11076819

[B17] Goslawski M. Piano M. R. Bian J. T. Church E. C. Szczurek M. Phillips S. A. (2013). Binge drinking impairs vascular function in young adults. J. Am. Coll. Cardiol. 62, 201–207. 10.1016/j.jacc.2013.03.049 23623907 PMC3727916

[B18] Green D. J. Spence A. Halliwill J. R. Cable N. T. Thijssen D. H. (2011). Exercise and vascular adaptation in asymptomatic humans. Exp. Physiol. 96, 57–70. 10.1113/expphysiol.2009.048694 20971800

[B19] Green D. J. Spence A. Rowley N. Thijssen D. H. Naylor L. H. (2012). Vascular adaptation in athletes: is there an 'athlete's artery'? Exp. Physiol. 97, 295–304. 10.1113/expphysiol.2011.058826 22179421

[B20] Hambrecht R. Adams V. Erbs S. Linke A. Kränkel N. Shu Y.. (2003). Regular physical activity improves endothelial function in patients with coronary artery disease by increasing phosphorylation of endothelial nitric oxide synthase. Circulation 107, 3152–3158. 10.1161/01.CIR.0000074229.93804.5C 12810615

[B21] Hamburg N. M. McMackin C. J. Huang A. L. Shenouda S. M. Widlansky M. E. Schulz E.. (2007). Physical inactivity rapidly induces insulin resistance and microvascular dysfunction in healthy volunteers. Arterioscler. Thromb. Vasc. Biol. 27, 2650–2656. 10.1161/ATVBAHA.107.153288 17932315 PMC2596308

[B22] Heiss C. Amabile N. Lee A. C. Real W. M. Schick S. F. Lao D.. (2008). Brief secondhand smoke exposure depresses endothelial progenitor cells activity and endothelial function: sustained vascular injury and blunted nitric oxide production. J. Am. Coll. Cardiol. 51, 1760–1771. 10.1016/j.jacc.2008.01.040 18452782

[B23] Higashi Y. Noma K. Yoshizumi M. Kihara Y. (2009). Endothelial function and oxidative stress in cardiovascular diseases. Circ. J. 73, 411–418. 10.1253/circj.CJ-08-1102 19194043

[B24] Jialal I. Devaraj S. (2009). The role of C-reactive protein activation of nuclear factor kappa-B in the pathogenesis of unstable angina. J. Am. Coll. Cardiol. 2007, 195–197. 10.1016/j.jacc.2006.10.018 17222730

[B25] Joyner M. J. Green D. J. (2009). Exercise protects the cardiovascular system: effects beyond traditional risk factors. J. Physiol. 587, 5551–5558. 10.1113/jphysiol.2009.179432 19736305 PMC2805367

[B26] Karatzi K. Papamichael C. Karatzis E. Papaioannou T. G. Voidonikola P. T. Vamvakou G. D.. (2008). Postprandial improvement of endothelial function by red wine and olive oil antioxidants: a synergistic effect of components of the Mediterranean diet. J. Am. Coll. Nutr. 27, 448–453. 10.1080/07315724.2008.10719724 18978163

[B27] Kawano H. Motoyama T. Hirashima O. Hirai N. Miyao Y. Sakamoto T.. (1999). Hyperglycemia rapidly suppresses flow-mediated endothelium-dependent vasodilation of brachial artery. J. Am. Coll. Cardiol. 34, 146–154. 10.1016/S0735-1097(99)00168-0 10400004

[B28] Kim F. Tysseling K. A. Rice J. Pham M. Haji L. Gallis B. M.. (2005). Free fatty acid impairment of nitric oxide production in endothelial cells is mediated by IKKbeta. Arterioscler. Thromb. Vasc. Biol. 25, 989–994. 10.1161/01.ATV.0000160549.60980.a8 15731493

[B29] Kingwell B. A. Sherrard B. Jennings G. L. Dart A. M. (1997). Four weeks of cycle training increases basal production of nitric oxide from the forearm. Am. J. Physiol. 272, H1070–1077. 10.1152/ajpheart.1997.272.3.H1070 9087577

[B30] Le Floch J. P. Escuyer P. Baudin E. Baudon D. Perlemuter L. (1990). Blood glucose area under the curve methodological aspects. Diabetes Care 13, 172–175. 10.2337/diacare.13.2.172 2351014

[B31] Leiter L. A. Ceriello A. Davidson J. A. Hanefeld M. Monnier L. Owens D. R.. (2005). Postprandial glucose regulation: new data and new implications. Clin. Ther. 27 (Suppl. B), S42–56. 10.1016/j.clinthera.2005.11.020 16519037

[B32] Mah E. Noh S. K. Ballard K. D. Matos M. E. Volek J. S. Bruno R. S. (2011). Postprandial hyperglycemia impairs vascular endothelial function in healthy men by inducing lipid peroxidation and increasing asymmetric dimethylarginine:arginine. J. Nutr. 141, 1961–1968. 10.3945/jn.111.144592 21940510

[B33] Mahmoud A. M. Szczurek M. R. Blackburn B. K. Mey J. T. Chen Z. Robinson A. T.. (2016). Hyperinsulinemia augments endothelin-1 protein expression and impairs vasodilation of human skeletal muscle arterioles. Physiol. Rep. 4:e12895. 10.14814/phy2.12895 27796268 PMC5002909

[B34] Matsuda M. DeFronzo R. A. (1999). Insulin sensitivity indices obtained from oral glucose tolerance testing: comparison with the euglycemic insulin clamp. Diabetes Care 22, 1462–1470. 10.2337/diacare.22.9.1462 10480510

[B35] Matthews D. R. Hosker J. P. Rudenski A. S. Naylor B. A. Treacher D. F. Turner R. C. (1985). Homeostasis model assessment: insulin resistance and beta-cell function from fasting plasma glucose and insulin concentrations in man. Diabetologia 28, 412–419. 10.1007/BF00280883 3899825

[B36] Padilla J. Harris R. A. Fly A. D. Rink L. D. Wallace J. P. (2006). The effect of acute exercise on endothelial function following a high-fat meal. Eur. J. Appl. Physiol. 98, 256–262. 10.1007/s00421-006-0272-z 16896723

[B37] Patel C. Ghanim H. Ravishankar S. Sia C. L. Viswanathan P. Mohanty P.. (2007). Prolonged reactive oxygen species generation and nuclear factor-kappaB activation after a high-fat, high-carbohydrate meal in the obese. J. Clin. Endocrinol. Metab. 92, 4476–4479. 10.1210/jc.2007-0778 17785362

[B38] Phillips S. A. Das E. Wang J. Pritchard K. Gutterman D. D. (2011). Resistance and aerobic exercise protects against acute endothelial impairment induced by a single exposure to hypertension during exertion. J. Appl. Physiol. 110, 1013–1020. 10.1152/japplphysiol.00438.2010 21252216 PMC3075126

[B39] Plotnick G. D. Corretti M. C. Vogel R. A. (1997). Effect of antioxidant vitamins on the transient impairment of endothelium-dependent brachial artery vasoactivity following a single high-fat meal. JAMA 278, 1682–1686. 10.1001/jama.1997.03550200058032 9388088

[B40] Pyke K. E. Tschakovsky M. E. (2007). Peak vs. total reactive hyperemia: which determines the magnitude of flow-mediated dilation? J. Appl. Physiol. 102, 1510–1519. 10.1152/japplphysiol.01024.2006 17170205

[B41] Robinson A. T. Fancher I. S. Mahmoud A. M. Phillips S. A. (2018). Microvascular vasodilator plasticity after acute exercise. Exerc. Sport Sci. Rev. 46, 48–55. 10.1249/JES.0000000000000130 28816705 PMC5735031

[B42] Robinson A. T. Fancher I. S. Sudhahar V. Bian J. T. Cook M. Mahmoud A. M. (2017). Short-term regular aerobic exercise reduces oxidative stress produced by acute high intraluminal pressure in the adipose microvasculature. Am. J. Physiol. Heart Circ. Physiol. 312, H896-H906. 10.1152/ajpheart.00684.2016 28235790 PMC5451589

[B43] Robinson A. T. Franklin N. C. Norkeviciute E. Bian J. T. Babana J. C. Szczurek M. R.. (2016). Improved arterial flow-mediated dilation after exertion involves hydrogen peroxide in overweight and obese adults following aerobic exercise training. J. Hypertens. 34, 1309–1316. 10.1097/HJH.0000000000000946 27137176

[B44] Rush J. W. Laughlin M. H. Woodman C. R. Price E. M. (2000). SOD-1 expression in pig coronary arterioles is increased by exercise training. Am. J. Physiol. Heart Circ. Physiol. 279, H2068–2076. 10.1152/ajpheart.2000.279.5.H2068 11045939

[B45] Steinberg H. O. Tarshoby M. Monestel R. Hook G. Cronin J. Johnson A.. (1997). Elevated circulating free fatty acid levels impair endothelium-dependent vasodilation. J. Clin. Invest. 100, 1230–1239. 10.1172/JCI119636 9276741 PMC508300

[B46] Thom N. J. Early A. R. Hunt B. E. Harris R. A. Herring M. P. (2016). Eating and arterial endothelial function: a meta-analysis of the acute effects of meal consumption on flow-mediated dilation. Obes. Rev. 17, 1080–1090. 10.1111/obr.12454 27469597

[B47] Tinken T. M. Thijssen D. H. Black M. A. Cable N. T. Green D. J. (2008). Time course of change in vasodilator function and capacity in response to exercise training in humans. J. Physiol. 586, 5003–5012. 10.1113/jphysiol.2008.158014 18755749 PMC2614057

[B48] Title L. M. Cummings P. M. Giddens K. Nassar B. A. (2000). Oral glucose loading acutely attenuates endothelium-dependent vasodilation in healthy adults without diabetes: an effect prevented by vitamins C and E. J. Am. Coll. Cardiol. 36, 2185–2191. 10.1016/S0735-1097(00)00980-3 11127459

[B49] Tushuizen M. E. Nieuwland R. Scheffer P. G. Sturk A. Heine R. J. Diamant M. (2006). Two consecutive high-fat meals affect endothelial-dependent vasodilation, oxidative stress and cellular microparticles in healthy men. J. Thromb. Haemost. 4, 1003–1010. 10.1111/j.1538-7836.2006.01914.x 16689751

[B50] Tyldum G. A. Schjerve I. E. Tjønna A. E. Kirkeby-Garstad I. Stølen T. O. Richardson R. S.. (2009). Endothelial dysfunction induced by post-prandial lipemia: complete protection afforded by high-intensity aerobic interval exercise. J. Am. Coll. Cardiol. 53, 200–206. 10.1016/j.jacc.2008.09.033 19130989 PMC2650775

[B51] Vogel R. A. Corretti M. C. Plotnick G. D. (1997). Effect of a single high-fat meal on endothelial function in healthy subjects. Am. J. Cardiol. 79, 350–354. 10.1016/S0002-9149(96)00760-6 9036757

[B52] Weiss E. P. Arif H. Villareal D. T. Marzetti E. Holloszy J. O. (2008). Endothelial function after high-sugar-food ingestion improves with endurance exercise performed on the previous day. Am. J. Clin. Nutr. 88, 51–57. 10.1093/ajcn/88.1.51 18614723 PMC2585377

[B53] Xiang G. D. Sun H. L. Zhao L. S. Hou J. Yue L. Xu L. (2008). The antioxidant alpha-lipoic acid improves endothelial dysfunction induced by acute hyperglycaemia during OGTT in impaired glucose tolerance. Clin. Endocrinol. (Oxf). 68, 716–723. 10.1111/j.1365-2265.2007.03099.x 18070144

[B54] Zilversmit D. B. (1979). Atherogenesis: a postprandial phenomenon. Circulation 60, 473–485. 10.1161/01.CIR.60.3.473 222498

